# 6-[(2-Hy­droxy­eth­yl)amino]-7*H*-dibenzo[*de*,*h*]quinolin-7-one

**DOI:** 10.1107/S1600536812024440

**Published:** 2012-06-02

**Authors:** Huang Tang, Zhi-Yu Wang, Yan-Cheng Liu

**Affiliations:** a State Key Laboratory Cultivation Base for the Chemistry and Molecular Engineering of Medicinal Resources, School of Chemistry & Chemical Engineering, Guangxi Normal University, Guilin 541004, People’s Republic of China

## Abstract

The title compound, C_18_H_14_N_2_O_2_, is a new oxoisoaporphine derivative synthesized by alkyl­ation of 6-chloro-1-aza­benzanthrone. The oxoisoaporphine fragment deviates significantly from planarity with a dihedral angle of 5.1 (1)° between the heterocycle and the remote benzene ring. The amino and oxo groups are involved in an intra­molecular N—H⋯O hydrogen bond, while the hy­droxy groups form inter­molecular O—H⋯N hydrogen bonds, which link pairs of mol­ecules into inversion dimers. In the dimer, two approximately parallel oxoisoaporphine fragments exhibit π–π inter­actions between the aromatic rings, the shortest centroid–centroid distance being 3.649 (3) Å.

## Related literature

For related oxoisoaporphine alkaloids, see: Tang *et al.* (2011 ▶, 2012 ▶). For background to the synthesis of 6-chloro-1-aza­benzanthrone, see: Iwashima *et al.* (1984 ▶).
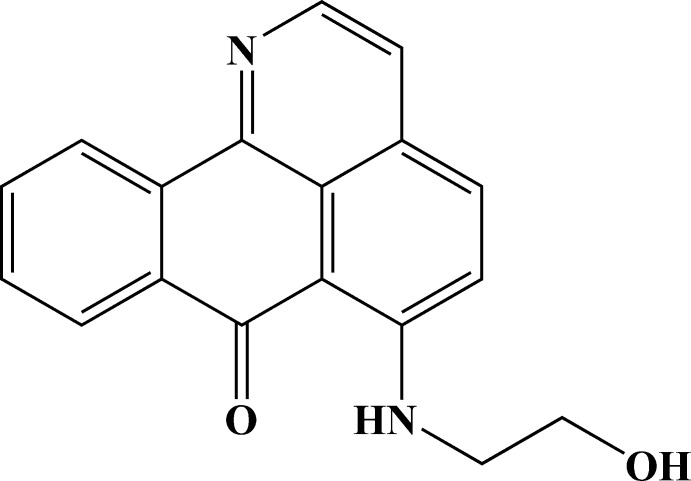


## Experimental

### 

#### Crystal data

C_18_H_14_N_2_O_2_
*M* *_r_* = 290.31
Monoclinic, 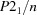

*a* = 9.8047 (16) Å
*b* = 12.865 (2) Å
*c* = 10.7623 (17) Å
β = 100.113 (2)°
*V* = 1336.4 (4) Å^3^
*Z* = 4
Mo *K*α radiation
μ = 0.10 mm^−1^
*T* = 296 K
0.37 × 0.23 × 0.16 mm

#### Data collection

Bruker APEXII CCD diffractometer
Absorption correction: multi-scan (*SADABS*; Sheldrick, 2007 ▶) *T*_min_ = 0.966, *T*_max_ = 0.985
6462 measured reflections
2359 independent reflections
1918 reflections with *I* > 2σ(*I*)
*R*_int_ = 0.023

#### Refinement

*R*[*F* ^2^ > 2σ(*F* ^2^)] = 0.041
*wR*(*F* ^2^) = 0.124
*S* = 1.06
2359 reflections
200 parameters
H-atom parameters constrained
Δρ_max_ = 0.16 e Å^−3^
Δρ_min_ = −0.18 e Å^−3^

### 

Data collection: *APEX2* (Bruker, 2007 ▶); cell refinement: *SAINT* (Bruker, 2007 ▶); data reduction: *SAINT*; program(s) used to solve structure: *SHELXS97* (Sheldrick, 2008 ▶); program(s) used to refine structure: *SHELXL97* (Sheldrick, 2008 ▶); molecular graphics: *SHELXTL* (Sheldrick, 2008 ▶); software used to prepare material for publication: *SHELXTL*.

## Supplementary Material

Crystal structure: contains datablock(s) I, global. DOI: 10.1107/S1600536812024440/cv5299sup1.cif

Structure factors: contains datablock(s) I. DOI: 10.1107/S1600536812024440/cv5299Isup2.hkl

Supplementary material file. DOI: 10.1107/S1600536812024440/cv5299Isup3.cml

Additional supplementary materials:  crystallographic information; 3D view; checkCIF report

## Figures and Tables

**Table 1:** Hydrogen-bond geometry (Å, °)

*D*—H⋯*A*	*D*—H	H⋯*A*	*D*⋯*A*	*D*—H⋯*A*
N2—H2*A*⋯O1	0.86	1.90	2.5990 (17)	138
O2—H2⋯N1^i^	0.82	2.24	3.0434 (18)	165

Symmetry code: (i) 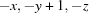.

**Fig. 1.**
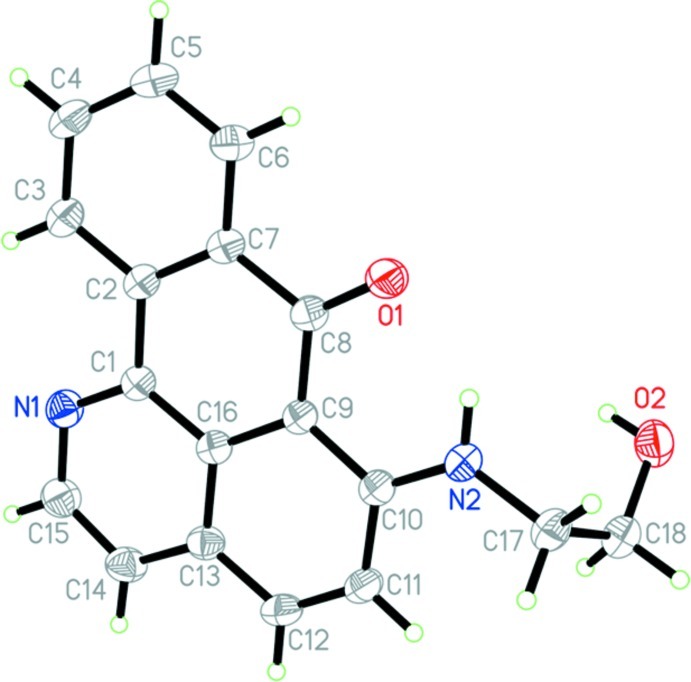
The molecular structure of (I) showing the atom-labelling scheme. Displacement ellipsoids are drawn at the 30% probability level.

**Fig. 2.**
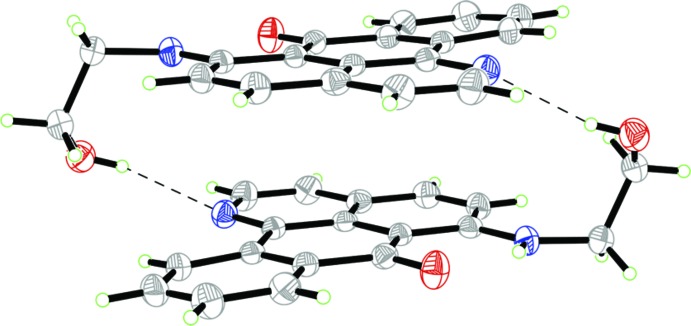
A hydrogen-bonded (dashed lines) dimer in (I).

## supplementary crystallographic information

### Comment

Oxoisoaporphine alkaloids are known due to their pharmaceutical activities, such as antitumoral and antidemential activities. As a continuation of our study of novel oxoisoaporphine-based inhibitors (Tang *et al.*, 2012), we present here the title compound (I), which is a new oxoisoaporphine synthesized by alkylation of 6-chloro-1-azabenzanthrone. It is structurally similar to the recently reported crystal structure of a new halogenated oxoisoaporphine by us, in which a chlorine atom replaced the 10- hydrogen atom but without substitution on the 6- position (Tang *et al.*, 2012).

In (I) (Fig. 1), the conjugated aromatic fragments of the oxoisoaporphine, including the heterocyclic isoquinoline and the remote benzene ring, is not entirely co-planar. The dihedral angle between the heterocycle of isoquinolinol and the remote benzene ring is 5.1 (1)°. The 2-hydroxyethylamino group on the 6-position of oxoisoaporphine plane is nearly vertical to the plane, with N2—C17—C18 bond angle of 113.18 (13)°. There exists intramolecular hydrogen bonds of N—H···O from the 7-carbonyl oxygen atom to the 6-imino group (–NH). Furthermore, every two oxoisoaporphines form a centrosymmetric dimer (Fig. 2), linked by intermolecular O—H···N hydrogen bonds (Table 1), in which N atom is the heterocyclic nitrogen atom of one oxoisoaporphine and O atom is the hydroxyethyl oxygen atom of another oxoisoaporphine. Each dimer is also stablized by π-π interaction between the two approximately parallel oxoisoaporphine fragments with the shortest centroid-centroid distance of 3.649 (3) Å.

### Experimental

6-Chloro-1-azabenzanthrone (3.0 mmol), ethanolamine (15 mmol), and NaI (0.1 g) were mixed in pentanol. The mixture was stirred and refluxed for 8 h, and then cooled at room temperature. The mixture was diluted with chloroform and made basic by 5% KOH solution. The organic layer was washed with water and brine and dried over anhydrous MgSO_4_. After concentration, the resulting residue was purified on silica gel chromatography (CHCl_3_:MeOH = 100:3) to give 6-(2-hydroxyethylamino)-7*H*-dibenzo[*de,h*]quinolin-7-one as reddish brown solid (yield 59%). Single crystals suitable for X-ray diffraction analysis were obtained by slow evaporation of a chloroform-ethanol solution (4:1 *v*/*v*). ^1^H NMR (CDCl_3_, 500 MHz)*δ*: 3.50 (br, 1H), 3.80 (dd, 2H, *J_1_*=6.4 Hz and *J_2_*=10.9 Hz), 4.11 (t, 2H,*J*=5.2 Hz), 7.38 (d, 1H,*J*=9.4 Hz,), 7.57 (d, 1H,*J*=5.2 Hz), 7.71 (t, 1H, *J*=7.6 Hz), 7.79 (d, 1H, *J*=9.4 H), 7.83 (t, 1H, *J*=7.6 Hz), 8.55 (d, 1H, *J*=8.0 Hz),8.70 (d, 1H, *J*=5.2 Hz), 9.01 (d, 1H, *J*=8.0 Hz), 12.02(s, 1H); ESI-MS *m/z*: 291 [*M*+H]^+^.

### Refinement

All H atoms were geometrically positioned (C—H 0.93–0.97 Å; O—H 0.82 Å; N—H 0.86 Å), and allowed to ride on their parent atoms, with *U*_iso_(H) = 1.2 –1.5 *U*_eq_(C, N, O).

### Crystal data

**Table:**

C_18_H_14_N_2_O_2_	*F*(000) = 608
*M**_r_* = 290.31	*D*_x_ = 1.443 Mg m^−^^3^
Monoclinic, *P*2_1_/*n*	Mo *K*α radiation, λ = 0.71073 Å
*a* = 9.8047 (16) Å	Cell parameters from 2722 reflections
*b* = 12.865 (2) Å	θ = 2.5–26.8°
*c* = 10.7623 (17) Å	µ = 0.10 mm^−^^1^
β = 100.113 (2)°	*T* = 296 K
*V* = 1336.4 (4) Å^3^	Rod, red
*Z* = 4	0.37 × 0.23 × 0.16 mm

### Data collection

**Table:**

Bruker APEXII CCD diffractometer	2359 independent reflections
Radiation source: fine-focus sealed tube	1918 reflections with *I* > 2σ(*I*)
Graphite monochromator	*R*_int_ = 0.023
φ and ω scans	θ_max_ = 25.1°, θ_min_ = 2.5°
Absorption correction: multi-scan (*SADABS*; Sheldrick, 2007)	*h* = −10→11
*T*_min_ = 0.966, *T*_max_ = 0.985	*k* = −15→15
6462 measured reflections	*l* = −12→9

### Refinement

**Table:**

Refinement on *F*^2^	Primary atom site location: structure-invariant direct methods
Least-squares matrix: full	Secondary atom site location: difference Fourier map
*R*[*F*^2^ > 2σ(*F*^2^)] = 0.041	Hydrogen site location: inferred from neighbouring sites
*wR*(*F*^2^) = 0.124	H-atom parameters constrained
*S* = 1.06	*w* = 1/[σ^2^(*F*_o_^2^) + (0.0673*P*)^2^ + 0.2157*P*] where *P* = (*F*_o_^2^ + 2*F*_c_^2^)/3
2359 reflections	(Δ/σ)_max_ < 0.001
200 parameters	Δρ_max_ = 0.16 e Å^−^^3^
0 restraints	Δρ_min_ = −0.18 e Å^−^^3^

### Special details

**Table:**

Geometry. All e.s.d.'s (except the e.s.d. in the dihedral angle between two l.s. planes) are estimated using the full covariance matrix. The cell e.s.d.'s are taken into account individually in the estimation of e.s.d.'s in distances, angles and torsion angles; correlations between e.s.d.'s in cell parameters are only used when they are defined by crystal symmetry. An approximate (isotropic) treatment of cell e.s.d.'s is used for estimating e.s.d.'s involving l.s. planes.
Refinement. Refinement of *F*^2^ against ALL reflections. The weighted *R*-factor *wR* and goodness of fit *S* are based on *F*^2^, conventional *R*-factors *R* are based on *F*, with *F* set to zero for negative *F*^2^. The threshold expression of *F*^2^ > σ(*F*^2^) is used only for calculating *R*-factors(gt) *etc*. and is not relevant to the choice of reflections for refinement. *R*-factors based on *F*^2^ are statistically about twice as large as those based on *F*, and *R*- factors based on ALL data will be even larger.

### Fractional atomic coordinates and isotropic or equivalent isotropic displacement parameters (Å^2^)

**Table:**

	*x*	*y*	*z*	*U*_iso_*/*U*_eq_	
N1	0.21519 (14)	0.40120 (11)	−0.17380 (12)	0.0495 (4)	
N2	0.12415 (13)	0.52517 (10)	0.35165 (12)	0.0458 (3)	
H2A	0.0651	0.4763	0.3552	0.055*	
O1	−0.01188 (13)	0.36243 (10)	0.25513 (11)	0.0577 (3)	
O2	−0.07301 (12)	0.66500 (10)	0.43621 (12)	0.0619 (4)	
H2	−0.0969	0.6421	0.3646	0.093*	
C1	0.16539 (15)	0.39304 (11)	−0.06593 (14)	0.0388 (3)	
C2	0.07454 (15)	0.30520 (11)	−0.05216 (14)	0.0392 (4)	
C3	0.04876 (17)	0.22647 (12)	−0.14357 (15)	0.0476 (4)	
H3	0.0925	0.2284	−0.2135	0.057*	
C4	−0.04056 (18)	0.14653 (13)	−0.13055 (18)	0.0561 (5)	
H4	−0.0573	0.0950	−0.1920	0.067*	
C5	−0.10582 (19)	0.14211 (14)	−0.02650 (18)	0.0588 (5)	
H5	−0.1679	0.0887	−0.0194	0.071*	
C6	−0.07916 (17)	0.21610 (13)	0.06584 (16)	0.0510 (4)	
H6	−0.1217	0.2118	0.1364	0.061*	
C7	0.01172 (15)	0.29820 (11)	0.05497 (14)	0.0399 (4)	
C8	0.04297 (15)	0.37373 (11)	0.15948 (14)	0.0407 (4)	
C9	0.13798 (14)	0.45744 (10)	0.14699 (13)	0.0368 (3)	
C10	0.17469 (14)	0.53226 (11)	0.24424 (14)	0.0396 (4)	
C11	0.26736 (16)	0.61437 (12)	0.22536 (16)	0.0468 (4)	
H11	0.2907	0.6641	0.2882	0.056*	
C12	0.32171 (16)	0.62173 (12)	0.11914 (16)	0.0494 (4)	
H12	0.3820	0.6760	0.1106	0.059*	
C13	0.28866 (15)	0.54778 (12)	0.01932 (15)	0.0434 (4)	
C14	0.34160 (18)	0.55268 (13)	−0.09252 (16)	0.0534 (4)	
H14	0.4035	0.6050	−0.1044	0.064*	
C15	0.30213 (18)	0.48015 (14)	−0.18484 (16)	0.0562 (5)	
H15	0.3374	0.4858	−0.2593	0.067*	
C16	0.19688 (14)	0.46567 (11)	0.03452 (13)	0.0374 (3)	
C17	0.15778 (18)	0.59083 (13)	0.46331 (15)	0.0505 (4)	
H17A	0.2545	0.6110	0.4737	0.061*	
H17B	0.1456	0.5509	0.5370	0.061*	
C18	0.06966 (18)	0.68764 (13)	0.45660 (17)	0.0532 (4)	
H18A	0.0940	0.7261	0.5349	0.064*	
H18B	0.0894	0.7315	0.3886	0.064*	

### Atomic displacement parameters (Å^2^)

**Table:**

	*U*^11^	*U*^22^	*U*^33^	*U*^12^	*U*^13^	*U*^23^
N1	0.0556 (8)	0.0503 (8)	0.0433 (8)	−0.0023 (6)	0.0104 (6)	−0.0005 (6)
N2	0.0481 (7)	0.0442 (7)	0.0446 (8)	−0.0034 (6)	0.0072 (6)	−0.0084 (6)
O1	0.0682 (8)	0.0605 (8)	0.0478 (7)	−0.0192 (6)	0.0193 (6)	−0.0059 (5)
O2	0.0572 (8)	0.0731 (9)	0.0567 (8)	0.0077 (6)	0.0140 (6)	−0.0070 (6)
C1	0.0393 (8)	0.0374 (7)	0.0381 (8)	0.0030 (6)	0.0019 (6)	0.0012 (6)
C2	0.0402 (8)	0.0349 (8)	0.0394 (8)	0.0036 (6)	−0.0015 (6)	0.0008 (6)
C3	0.0525 (9)	0.0430 (9)	0.0449 (9)	0.0018 (7)	0.0019 (7)	−0.0049 (7)
C4	0.0608 (11)	0.0433 (9)	0.0598 (11)	−0.0049 (8)	−0.0016 (8)	−0.0127 (8)
C5	0.0591 (11)	0.0458 (9)	0.0690 (12)	−0.0170 (8)	0.0046 (9)	−0.0057 (9)
C6	0.0518 (10)	0.0472 (9)	0.0530 (10)	−0.0097 (7)	0.0064 (7)	0.0011 (8)
C7	0.0400 (8)	0.0354 (8)	0.0418 (8)	0.0003 (6)	0.0002 (6)	0.0015 (6)
C8	0.0407 (8)	0.0400 (8)	0.0399 (8)	−0.0004 (6)	0.0031 (6)	0.0011 (6)
C9	0.0355 (7)	0.0348 (7)	0.0384 (8)	0.0027 (6)	0.0013 (6)	−0.0004 (6)
C10	0.0361 (7)	0.0381 (8)	0.0424 (8)	0.0052 (6)	0.0005 (6)	−0.0011 (6)
C11	0.0471 (9)	0.0406 (8)	0.0501 (9)	−0.0036 (7)	0.0012 (7)	−0.0089 (7)
C12	0.0468 (9)	0.0411 (9)	0.0585 (11)	−0.0104 (7)	0.0047 (7)	−0.0018 (7)
C13	0.0413 (8)	0.0399 (8)	0.0474 (9)	−0.0015 (6)	0.0037 (6)	0.0032 (7)
C14	0.0543 (10)	0.0533 (10)	0.0535 (10)	−0.0118 (8)	0.0121 (8)	0.0049 (8)
C15	0.0627 (10)	0.0601 (11)	0.0485 (10)	−0.0084 (8)	0.0171 (8)	0.0032 (8)
C16	0.0355 (7)	0.0340 (7)	0.0403 (8)	0.0041 (6)	0.0002 (6)	0.0038 (6)
C17	0.0547 (9)	0.0513 (9)	0.0430 (9)	0.0012 (7)	0.0016 (7)	−0.0062 (7)
C18	0.0599 (10)	0.0487 (9)	0.0503 (10)	0.0018 (7)	0.0081 (8)	−0.0074 (8)

### Geometric parameters (Å, º)

**Table:**

N1—C1	1.340 (2)	C7—C8	1.477 (2)
N1—C15	1.345 (2)	C8—C9	1.446 (2)
N2—C10	1.338 (2)	C9—C10	1.421 (2)
N2—C17	1.458 (2)	C9—C16	1.434 (2)
N2—H2A	0.8600	C10—C11	1.431 (2)
O1—C8	1.2508 (18)	C11—C12	1.347 (2)
O2—C18	1.408 (2)	C11—H11	0.9300
O2—H2	0.8200	C12—C13	1.429 (2)
C1—C16	1.421 (2)	C12—H12	0.9300
C1—C2	1.462 (2)	C13—C14	1.393 (2)
C2—C7	1.402 (2)	C13—C16	1.416 (2)
C2—C3	1.404 (2)	C14—C15	1.368 (2)
C3—C4	1.374 (2)	C14—H14	0.9300
C3—H3	0.9300	C15—H15	0.9300
C4—C5	1.385 (3)	C17—C18	1.510 (2)
C4—H4	0.9300	C17—H17A	0.9700
C5—C6	1.368 (2)	C17—H17B	0.9700
C5—H5	0.9300	C18—H18A	0.9700
C6—C7	1.400 (2)	C18—H18B	0.9700
C6—H6	0.9300		
			
C1—N1—C15	117.96 (14)	N2—C10—C11	120.90 (13)
C10—N2—C17	127.43 (14)	C9—C10—C11	118.72 (14)
C10—N2—H2A	116.3	C12—C11—C10	121.88 (14)
C17—N2—H2A	116.3	C12—C11—H11	119.1
C18—O2—H2	109.5	C10—C11—H11	119.1
N1—C1—C16	122.94 (14)	C11—C12—C13	121.34 (14)
N1—C1—C2	117.99 (13)	C11—C12—H12	119.3
C16—C1—C2	119.06 (13)	C13—C12—H12	119.3
C7—C2—C3	118.62 (14)	C14—C13—C16	118.32 (14)
C7—C2—C1	119.75 (13)	C14—C13—C12	123.34 (14)
C3—C2—C1	121.63 (14)	C16—C13—C12	118.34 (14)
C4—C3—C2	120.55 (16)	C15—C14—C13	119.71 (15)
C4—C3—H3	119.7	C15—C14—H14	120.1
C2—C3—H3	119.7	C13—C14—H14	120.1
C3—C4—C5	120.43 (15)	N1—C15—C14	123.62 (16)
C3—C4—H4	119.8	N1—C15—H15	118.2
C5—C4—H4	119.8	C14—C15—H15	118.2
C6—C5—C4	120.16 (16)	C13—C16—C1	117.40 (14)
C6—C5—H5	119.9	C13—C16—C9	120.74 (13)
C4—C5—H5	119.9	C1—C16—C9	121.86 (13)
C5—C6—C7	120.50 (16)	N2—C17—C18	113.18 (13)
C5—C6—H6	119.8	N2—C17—H17A	108.9
C7—C6—H6	119.8	C18—C17—H17A	108.9
C6—C7—C2	119.68 (14)	N2—C17—H17B	108.9
C6—C7—C8	118.86 (14)	C18—C17—H17B	108.9
C2—C7—C8	121.43 (13)	H17A—C17—H17B	107.8
O1—C8—C9	122.71 (14)	O2—C18—C17	112.42 (14)
O1—C8—C7	119.24 (13)	O2—C18—H18A	109.1
C9—C8—C7	118.04 (13)	C17—C18—H18A	109.1
C10—C9—C16	118.97 (13)	O2—C18—H18B	109.1
C10—C9—C8	121.34 (14)	C17—C18—H18B	109.1
C16—C9—C8	119.69 (13)	H18A—C18—H18B	107.9
N2—C10—C9	120.38 (13)		
			
C15—N1—C1—C16	2.1 (2)	C16—C9—C10—N2	−178.32 (12)
C15—N1—C1—C2	−177.97 (14)	C8—C9—C10—N2	1.6 (2)
N1—C1—C2—C7	−175.08 (13)	C16—C9—C10—C11	1.1 (2)
C16—C1—C2—C7	4.8 (2)	C8—C9—C10—C11	−178.90 (13)
N1—C1—C2—C3	5.3 (2)	N2—C10—C11—C12	178.58 (14)
C16—C1—C2—C3	−174.74 (13)	C9—C10—C11—C12	−0.9 (2)
C7—C2—C3—C4	2.6 (2)	C10—C11—C12—C13	0.4 (2)
C1—C2—C3—C4	−177.84 (14)	C11—C12—C13—C14	179.55 (16)
C2—C3—C4—C5	−0.4 (3)	C11—C12—C13—C16	−0.1 (2)
C3—C4—C5—C6	−1.6 (3)	C16—C13—C14—C15	1.2 (2)
C4—C5—C6—C7	1.4 (3)	C12—C13—C14—C15	−178.41 (15)
C5—C6—C7—C2	0.8 (2)	C1—N1—C15—C14	−0.4 (3)
C5—C6—C7—C8	−177.12 (14)	C13—C14—C15—N1	−1.2 (3)
C3—C2—C7—C6	−2.7 (2)	C14—C13—C16—C1	0.3 (2)
C1—C2—C7—C6	177.68 (13)	C12—C13—C16—C1	179.97 (12)
C3—C2—C7—C8	175.10 (13)	C14—C13—C16—C9	−179.27 (13)
C1—C2—C7—C8	−4.5 (2)	C12—C13—C16—C9	0.4 (2)
C6—C7—C8—O1	0.3 (2)	N1—C1—C16—C13	−2.0 (2)
C2—C7—C8—O1	−177.57 (13)	C2—C1—C16—C13	178.04 (12)
C6—C7—C8—C9	179.47 (13)	N1—C1—C16—C9	177.53 (13)
C2—C7—C8—C9	1.6 (2)	C2—C1—C16—C9	−2.4 (2)
O1—C8—C9—C10	0.1 (2)	C10—C9—C16—C13	−0.9 (2)
C7—C8—C9—C10	−179.09 (12)	C8—C9—C16—C13	179.11 (12)
O1—C8—C9—C16	−179.94 (13)	C10—C9—C16—C1	179.52 (12)
C7—C8—C9—C16	0.9 (2)	C8—C9—C16—C1	−0.4 (2)
C17—N2—C10—C9	175.77 (13)	C10—N2—C17—C18	86.77 (19)
C17—N2—C10—C11	−3.7 (2)	N2—C17—C18—O2	56.18 (19)

### Hydrogen-bond geometry (Å, º)

**Table:**

*D*—H···*A*	*D*—H	H···*A*	*D*···*A*	*D*—H···*A*
N2—H2*A*···O1	0.86	1.90	2.5990 (17)	138
O2—H2···N1^i^	0.82	2.24	3.0434 (18)	165

Symmetry code: (i) −*x*, −*y*+1, −*z*.
